# Phase I/II study of temsirolimus for patients with unresectable Hepatocellular Carcinoma (HCC)- a correlative study to explore potential biomarkers for response

**DOI:** 10.1186/s12885-015-1334-6

**Published:** 2015-05-12

**Authors:** Winnie Yeo, Stephen L Chan, Frankie KF Mo, Cheuk M Chu, Joyce WY Hui, Joanne HM Tong, Anthony WH Chan, Jane Koh, Edwin P Hui, Herbert Loong, Kirsty Lee, Leung Li, Brigette Ma, Ka F To, Simon CH Yu

**Affiliations:** 1 Comprehensive Cancer Trials Unit, Department of Clinical Oncology, State Key Lab in Oncology in South China, Prince of Wales Hospital, Chinese University of Hong Kong, Shatin, Hong Kong; 2 Department of Diagnostic and Interventional Radiology, Prince of Wales Hospital, Chinese University of Hong Kong, Shatin, Hong Kong; 3 Department of Anatomical and Cellular Pathology, Prince of Wales Hospital, Chinese University of Hong Kong, Shatin, Hong Kong

**Keywords:** mTOR inhibitor, Liver cancer, Palliative

## Abstract

**Background:**

The oncogenic PI3K/Akt/mTOR pathway is frequently activated in HCC. Data on the mTOR inhibitor, temsirolimus, is limited in HCC patients with concomitant chronic liver disease. The objectives of this study were: (1) In phase I, to determine DLTs and MTD of temsirolimus in HCC patients with chronic liver disease; (2) In phase II, to assess activity of temsirolimus in HCC, and (3) to explore potential biomarkers for response.

**Methods:**

Major eligibility criteria included histologically confirmed advanced HCC and adequate organ function. In Phase I part of the study, temsirolimus was given weekly in 3-weekly cycle; dose levels were 20 mg (level 1), 25 mg (level 2) and 30 mg (level 3). The MTD was used in the subsequent phase II part; the primary endpoint was PFS and secondary endpoints were response and OS. In addition, exploratory analysis was conducted on pre-treatment tumour tissues to determine stathmin, pS6, pMTOR or p-AKT expressions as potential biomarkers for response. Overall survival and PFS were calculated using the Kaplan-Meier method. Reassessment CT scans were done every 6 weeks. All adverse events were reported using CTCAE v3.

**Results:**

The Phase I part consisted of 19 patients, 2 of 6 patients at level 3 experienced DLT; dose level 2 was determined to be the MTD. The phase II part consisted of 36 patients. Amongst 35 assessable patients, there were 1 PR, 20 SD and 14 PD. Overall, the median PFS was 2.83 months (95% C.I. 1.63-5.24). The median OS was 8.89 months (95% C.I. 5.89-13.30). Grade ≥ 3 that occurred in > 10% of patients included thrombocytopenia (4) and hyponatraemia (4). Exploratory analysis revealed that disease stabilization (defined as CR + PR + SD > 12 weeks) in tumours having high and low pMTOR H-scores to be 70% and 29% respectively (OR 5.667, 95% CI 1.129-28.454, p = 0.035).

**Conclusions:**

In HCC patients with chronic liver disease, the MTD of temsirolimus was 25 mg weekly in a 3-week cycle. The targeted PFS endpoint was not reached. However, further studies to identify appropriate patient subgroup are warranted.

**Trial registration:**

This study has been registered in ClinicalTrials.gov (Id: NCT00321594) on 1 December 2010.

## Background

Hepatocellular carcinoma (HCC) is the sixth most common cancer globally, and the third leading cause of cancer mortality both in Hong Kong and worldwide [1,2]. The outlook of patients with unresectable HCC is poor. To date, the only systemic agent that has been shown to provide survival benefit is sorafenib [3,4]. In parts of the world including Hong Kong, HCC patients often present with advanced disease stage, but the use of sorafenib has only been approved in recent years as standard therapy.

It has been well-established that numerous genetic abnormalities are involved in HCC; comprehensive genomic analyses shows that components of the phosphatidylinositol-3 kinase (PI3K)/Akt/mTOR pathway are dysregulated in 40-50% of HCC [5-7]. On the other hand, a meta-analysis of over 450 patients with HCC who received liver transplant demonstrated lower rates of recurrence and mortality for patients who received the mTOR inhibitor (mTORI), sirolimus, for immunosuppression [8]. The expansion of mTORIs as a therapeutic strategy for HCC was also strengthened by their successes in other cancers [9-12]. In various HCC models, mTORIs significantly reduced tumour volume and angiogenesis, delayed tumour growth and increased survival [5,6,13-16].

Everolimus had initially been evaluated in HCC in phase I and II studies. A US study achieved an MTD of 10 mg/day [17]; among the 25 patients enrolled, 10 achieved stable disease, one achieved partial response, and median survival was 8.4 months. In another study, Taiwanese patients tolerated only a daily dose of 7.5 mg, and the median survival was 7.7 months [18]. However, the efficacy of everolimus in HCC has not been confirmed by the recently reported global phase III study (EVOLVE-1, NCT01035229) [19].

Temsirolimus is a prodrug of sirolimus; it is administered intravenously and has a long half-life of 73 hours. To date, there has been limited clinical data on the use of temsirolimus in HCC patients who often suffer from chronic liver disease. We conducted a phase I/II study of temsirolimus (Torisel®) in patients with unresectable HCC, majority of whom had concomitant hepatitis B virus-related chronic liver disease. The objectives in the phase I study were to determine dose limiting toxicity (DLT) and maximum tolerated dose (MTD). Once the MTD was determined, the phase II portion of the study was conducted to determine the activity of temsirolimus.

Although promising results have been shown with temsirolimus in a number of malignancies, there has been very limited data on potential biomarkers that could enable appropriate selection of tumours which are likely to undergo a favorable clinical response. Further, the failure to demonstrate efficacy of everolimus in the EVOLVE study has highlighted the potential importance of appropriate patient selection. Thus, in the current study, an exploratory analysis was also conducted to determine if the expression of stathmin, pS6, pMTOR and p-AKT might be predictive for response to temsirolimus in HCC.

## Methods

Eligibility criteria included: Histologically/cytologically confirmed unresectable HCC; ECOG ≤2; measurable disease; life expectancy > 12 weeks; absolute neutrophil count ≥ 1.5 × 10^9^/L, platelets ≥ 80 × 10^9^/l, serum creatinine ≤ 150 μmol/L, total bilirubin ≤ 30 umol/l, albumin ≥ 28 g/l, alanine transaminases ≤ 5.0 × UNL (institutional upper normal limit), alkaline phosphatase ≤ 6 × UNL, prothrombin time ≤ 4 sec of ULN, and absence of clinical ascites.

The main exclusion criteria were Child’s B or C cirrhosis, use of other systemic treatments within 3 weeks prior to study entry; prior use of mTORI; significant cardiovascular disease; severe impairment of lung function; poorly controlled diabetes mellitus; and ≥ grade 2 pre-existing neuropathy.

Written consent was sought from individual patient to participate in the study and for the exploratory analysis that involved the use of tissue obtained for diagnostic purpose**.** This study was approved by the Clinical Research Ethics Committee of the Joint NTEC-Review Board of the Chinese University of Hong Kong, and has been registered in ClinicalTrials.gov (Id: NCT00321594).

### Pretreatment evaluation

All patients underwent complete medical history and physical examination, blood profiles including complete blood counts, renal and liver functions, fasting glucose and lipids, clotting profiles, alpha-fetoprotein (AFP), and hepatitis B surface antigen (HBsAg), hepatitis C antibody (anti-HCV), chest x-ray and CT scan of abdomen and/or other disease sites were performed.

### Treatment plan

Temsirolimus was added to 250 mL of 0.9% sodium chloride and administered intravenously over 30 minutes weekly, every 3 weeks. All patients received premedication with diphenhydramine 25 mg or 50 mg IV bolus dose 30 minutes prior to temsirolimus. Standard anti-emetics included at least a 5-HT3 antagonist. Patients who were HBsAg seropositive were also given lamivudine prior to study treatment.

### Phase I study

For the phase 1 study, there were 5 dose levels of temsirolimus: 10 (level −2), 15 (level −1), 20 (level 1), 25 (level 2) and 30 mg/week (level 3). Level 1 was the starting dose level.

DLT was defined during cycle 1 as: any grade 4 hematological toxicity; grade ≥3 non-hematological toxicity (excluding alopecia); grade 3 nausea, vomiting, or diarrhoea that did not respond to therapy; and treatment delay > 2 weeks.

The conventional 3 + 3 design was employed. Dose escalation was based on the modified Fibonacci method [20]. The MTD was defined as the dose below which ≥ 2 of 3 or ≥ 2 of 6 patients experiencing DLT. A total of 10 patients were entered into the MTD to further define toxicity.

### Treatment delay and modification

For each cycle, treatment was delayed if the ANC was <1.5 × 10^9^/L or platelet count was < 75 × 10^9^/ml on the scheduled day of drug administration. Patients who experienced grade 3 non-haematological toxicity, thrombocytopenia or febrile neutropenia, as well as grade 4 neutropenia continued to receive temsirolimus at the next lower dose level upon resolution of all toxicities to grade 1. For an individual, there could be a limit of two dose de-escalations for serious toxicity. The drug was discontinued for toxicities of the following nature: grade 4 non-hematological toxicities, thrombocytopenia/febrile neutropenia/recurrent grade 4 neutropenia despite dose reduction, as well as any haematological or non-haematological toxicity requiring interruption for ≥ 3 weeks.

Treatment was continued provided that toxicities were tolerable or until one of the following criteria applied: disease progression; intercurrent illness that prevented further treatment administration; unacceptable adverse events; patient’s decision; or investigator’s judgment.

### Phase II study

Upon determination of MTD, patients were enrolled into the phase II part of the trial at MTD; the 10 patients at the MTD in phase I were included in the phase II analysis.

### Definitions of response and toxicity

Tumour response assessment with CT every two cycles was assessed according to the Response Evaluation Criteria in Solid Tumors (RECIST) Committee [21]. Toxicity was graded according to Common Toxicity Criteria of the National Cancer Institute (NCI-CTC v3).

### Methodology for stathmin, pS6, pMTOR and p-AKT immunohistochemistry

Thirty-four patients had pre-treatment tissues available for this analysis. For immunohistochemistry, 5-μm tissue sections were prepared from each block. Tissue sections were deparaffinized, rehydrated and rinsed in distilled water. Antigen retrieval was done by using pressure cooker with 10 nM citrate buffer (pH 6.0) for 25 minutes. The endogenous peroxidase activity was then blocked by incubating the slides in 3% hydrogen peroxide in methanol for 10 min. The primary antibodies used in this study were STMN1 (1:50), pS6 (Ser235/236, 1:100), pMTOR (Ser2448, 1:50) and p-AKT (Ser473, clone D9E, 1:25) from Cell Signaling Technology (Danvers MA). The primary antibodies were incubated at 4°C overnight and chromogen development was performed using the DAKO EnVision System (Glostrup, Denmark) except for p-AKT, which was detected using the OptiView DAB IHC Detection Kit (Ventana Medical Systems).

An intensity score of 0 to 3 was assigned for the intensity of tumour cells (0, none; 1, weak; 2, intermediate; 3, strong). A proportional score was given by the estimated proportion of positive tumour cells in percentage. To assess the average degree of staining within a tumour, multiple regions were analyzed, and at least 100 tumour cells were assessed. The cytoplasmic expression was assessed by H-score system [22]. The formula for the H-score is: Histoscore = ∑(I × Pi), where I = intensity of staining and Pi = percentage of stained tumour cells, producing a cytoplasmic score ranging from 0 to 300. The scoring was independently assessed by two assessors (AWHC and JHMT) who were not aware of the clinical outcomes.

### Statistical methods

For the Phase I portion, the estimated patient number would be 14–19. For the phase II portion, the primary endpoint was progression free survival (PFS). The secondary endpoints were response according to RECIST, overall survival (OS) and toxicity. The PFS was assessed from day 1 of treatment cycle 1 to the date when objective disease progression was observed. OS was calculated from day 1 of treatment cycle 1 to the date of death. Death was regarded as a progression event in those subjects who died before disease progression. Subjects without documented objective progression at the time of the final analysis were censored at the date of their last tumour assessment. Survival curves were constructed using the Kaplan–Meier method.

The planned accrual for phase II was 30 assessable patients. Patients are considered assessable if they have completed ≥ 1 cycle of treatment or are removed from study due to disease progression. If the PFS at 3 months is ≤ 0.5, the regimen would be considered inactive. If the PFS at 3 months is ≥ 0.66, this regimen would be considered worthy of further investigation. If ≥ 18 of 30 assessable patients are observed to be progression-free by 3 months, the study would have 80% power and 0.18 significance level. An additional 6 patients (i.e. 20%) would be accrued to account for ineligibility, cancellation, major treatment violation, or other reasons. Therefore, the maximum accrual would be 36 patients (including the 10 patients from phase I at MTD). In order to observe enough events for the study, all patients would be followed up for at least 3 months.

Exploratory analysis on cytoplasmic expression of the biomarkers was viewed as hypothesis generating. The optimal cutoff for stathmin, pS6, pMTOR and p-AKT was determined by the receiver operating characteristic (ROC) curve distribution analysis [23,24]. Out of a total H-score of 300, the threshold for differentiating between positive and negative immunostaining were set at H-scores of 15, 120, 20 and 5 respectively; tumours were categorized as ‘low H-score’ and ‘high H-score’ depending on whether the individual score were ‘lower than or equal to’ or ‘higher than’ the respective thresholds. Response rates in terms of disease stabilization (defined as complete response [CR] + partial response [PR] + stable disease [SD] ≥ 12 weeks) and AFP drop in association with H-scores of stathmin, pS6, pMTOR and p-AKT cytoplasmic were compared using Fisher’s exact and proportional hazard model where applicable. Response assessment based on AFP was conducted for patients whose baseline AFP > 20 ng/ml and who had 2 cycles of study treatment. The drop in AFP based on baseline AFP was compared with the lowest level of AFP detected after 2 cycles of study treatment, and AFP response was defined as a > 20% decrease in AFP value [25].

## Results

From November 2009 to December 2011, a total of 45 patients were consented and entered.

### Phase I study

#### Patient characteristics and study drug dosing

Nineteen patients were entered, 3 in level 1, 10 in level 2 and 6 in level 3 (Table 1). The median age was 56.0 years (range 36–77). Fifteen (79%) were male, 14 (78%) had ECOG 0. Fifteen (79%) had chronic HBV and 1 was hepatitis C seropositive.

**Table 1:** **Summary of dose level and dose-limiting toxicities in phase 1**

Patient no.	Dose level	Dose-limiting toxicities
001	1	nil
002	1	nil
003	1	nil
004	2	nil
005	2	nil
006	2	nil
007	3	nil
008	3	Grade 3 syncope
009	3	nil
010	3	nil
011	3	nil
012	3	Treatment delay for > 2 weeks due to prolonged neutropenia
013	2	nil
014	2	nil
015	2	nil
016	2	nil
017	2	nil
018	2	nil
019	2	nil

Two out of 6 patients developed DLTs at level 3 (dose being 30 mg/week), including 1 who developed grade 3 syncope and 1 who had treatment delay for > 2 weeks due to prolonged neutropenia. Temsirolimus dose of 25 mg/week was declared as the MTD and the recommended phase II dose; at the MTD, temsirolimus was well tolerated with no DLTs. The 10 patients enrolled into the phase I study at MTD were included in the phase II analysis.

### Phase II study

#### Patient characteristics

The following analyses pertain to the 36 patients who were being enrolled into the phase II study.

Patient characteristics are shown in Table 2. Of note, 27 patients had BCLC stage C [26], 9 had BCLC stage B (including 8 who failed multiple lines of loco-regional therapies and 1 who had extensive intrahepatic disease); 24 (66.7%) had vascular involvement and 21 (58.3%) had extrahepatic metastases. Twenty-nine patients (80.5%) had received prior treatment for HCC; 13 (36.1%) had received ≥1 line of prior systemic therapies; 10 of the latter had received anti-vascular endothelial growth factor tyrosine kinase inhibitors (anti-VEGF TKIs). The median number of cycles was 3.5 (range: 1–16). Twelve (34%) patients underwent at least 6 cycles of temsirolimus. The follow-up data was frozen on 31 December 2013. The median follow-up was 8.89 months (95% C.I. 5.89-13.30). At the time of data cutoff, all patients had died; 34 (94.4%) were due to progressive disease, 1 due to liver failure and another due to pneumonia.

**Table 2:** **Baseline patient characteristics in phase II study**

Characteristic	No. of patients	%
No. of patients	36	100
Gender		
Male	31	86.1
Female	5	13.9
Age, years		
Median	56	
Range	26-77	
ECOG performance status		
0	24	66.7
1	12	33.3
Hepatitis status		
Hepatitis B	29	80.5
Hepatitis C	1	2.8
Non-B non-C	6	16.7
Baseline AFP > 10 μg/l		
Yes	25	69.4
No	11	30.6
Tumour Burden		
BCLC Stage B	28	
BCLC stage C	8	
Macroscopic vascular invasion	24	66.7
Extrahepatic disease	21	58.3
Prior therapy for HCC of any forms	29	80.6
Blood parameters (median, range):		
Total bilirubin	16 (5–34) umol/l	
Albumin	39. (32–48) g/l	
Alanine transaminase	40 (18–140) iu/l	
Alkaline phosphatase	108 (52–434) iu/l	
AFP	82 (1–118712) ug/l	
INR	1.06 (0.89-1.26)	
Creatinine	82 (44–136) umol/l	
Glucose	5.4 (4.0-8.5) mmol/l	
Triglyceride	0.9 (0.5-2.0) mmol/l	
LDL cholesterol	2.65 (1.6-7.3) mmol/l	
HDL cholesterol	1.15 (0.7-2.5) mmol/l	
Total cholesterol	4.45 (3.0-8.9) mmol/l	
Prior systemic therapy		
1 line	11	30.5
2 lines	1	2.8
3 lines	1	2.8
Prior local +/− regional therapy		
Surgery	23	63.9
*Local ablation	4	11.0
Transarterial therapy	20	55.5

*2 had radiofrequency ablation and 2 had percutaneous ethanol injection.

#### Response and survival

One patient was not assessable for response as he went abroad after receiving cycle 1 week 1 of temsirolimus. Amongst the 35 assessable patients, the best responses were: 1 PR (3%), 20 SD (57%) and 14 progressive disease [PD] (40.0%); 40% had disease stabilization.

**Figure 1:**
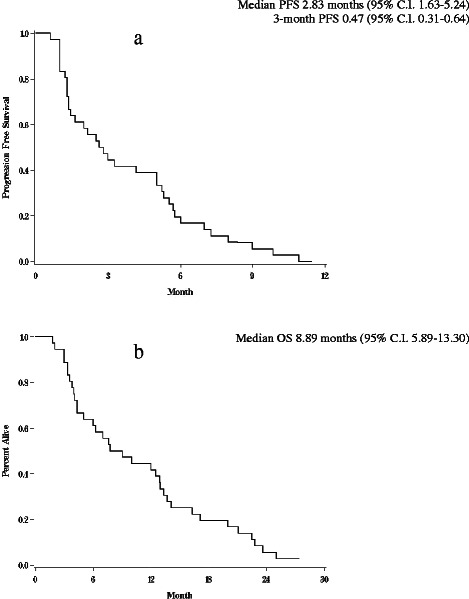
**(a)** Progression-free survival; **(b)** Overall survival of patients in the phase II study.

Overall, the median PFS was 2.83 months (95% C.I. 1.63-5.24); the 3-month PFS was 0.47 (95% C.I. 0.31-0.64) (Figure 1a). The median OS was 8.89 months (95% C.I. 5.89-13.30) (Figure 1b).

Unplanned exploratory analyses revealed that patients who received prior anti-VEGF TKIs had similar PFS and OS compared with those who did not. In addition, treatment outcome was not associated with viral etiologies (data not shown).

#### Toxicity

In the phase II portion study, toxicity was assessable in the 35 patients (Table 3). The most common adverse events that occurred in > 30% of patients included oral mucositis, rash, fatigue, cough, non-neutropenic fever, anorexia, insomnia, diarrhea, thrombocytopenia, and pain in abdomen and head. Grade ≥ 3 events that occurred in > 10% included hyponatraemia and thrombocytopenia.

**Table 3:** **Haematological and non-haematological toxicities according to the NCI CTC (version 3.0) (n = 35)**

	Worst grade (number of patients)		
Toxicities	1-2	3	4
Mucositis- oral	26	1	0
Rash	20	0	0
Fatigue	17	1	0
Cough	15	1	0
Fever	14	0	0
Anorexia	13	0	0
Pulmonary-Other	13	0	0
Insomnia	12	0	0
Pain- head	12	0	0
Pain- abdomen	10	1	0
Haemorrhage, nose	10	0	0
Oedema- limb	10	0	0
Pruritus	10	0	0
Gastrointestinal-Other	9	0	0
Diarrhoea	8	3	0
Dysphagia	8	1	0
Nausea	8	0	0
Pain- others	8	0	0
Platelets	7	4	0
Dyspnoea	7	1	0
Constipation	7	0	0
Distension	7	0	0
Dry mouth	7	0	0
Haemorrhage, other	6	0	0
Rigors/chills	6	0	0
Vomiting	6	0	0
Hyperglycaemia	5	1	0
Dizziness	5	0	0
Dry skin	5	0	0
Musculoskeletal-Other	5	0	0
Pain- muscle	5	0	0
Taste alteration	5	0	0
Hypokalaemia	4	1	1
Hemorrhoids	4	1	0
Hyponatraemia	0	4	0
Ascites	4	0	0
Infection- others with normal neutrophil counts	4	0	0
Alanine transaminase	3	2	0
Hyperbilirubinaemia	1	2	1
Infection- upper airway with normal neutrophil counts	1	3	0

Of note, hyperglycaemia occurred in 6 patients (17%; 4 grade 1–2 and 1 grade 3), while 1 patient developed grade 2 hypercholesterolaemia; all could be managed with standard medical therapies. Two patients developed interstitial pneumonitis, which resolved with corticosteroid and discontinuation of temsirolimus.

#### Exploratory analysis

Of the 35 assessable patients, 34 had pre-treatment tumour tissues available for this analysis, there were 14 patients who achieved disease stabilization.

**Figure 2:**
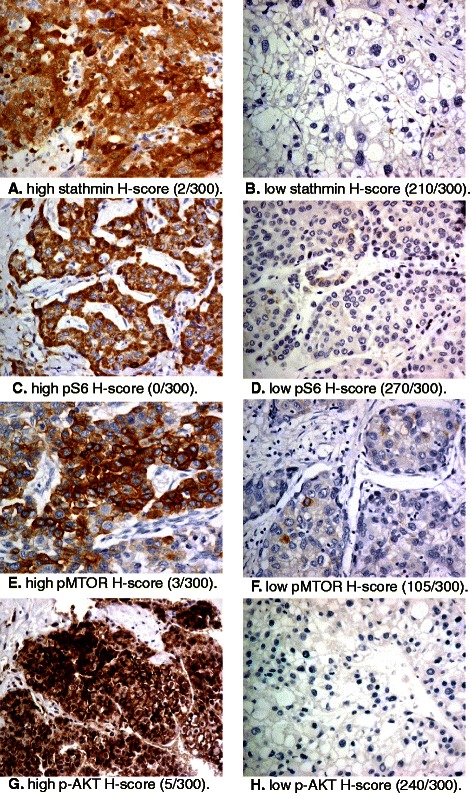
Immunohistochemical staining of pretreatment tumour tissues. **A**. high stathmin H-score (2/300). **B**. low stathmin H-score (210/300). **C**. high pS6 H-score (0/300). **D**. low pS6 H-score (270/300). **E**. high pMTOR H-score (3/300). **F**. low pMTOR H-score (105/300). **G**. high p-AKT H-score (5/300). **H**. low p-AKT H-score (240/300).

The H-scores for stathmin, pS6, pMTOR and pAKT of individual patient’s tumour are listed in Table 4. The immunohistochemical findings with respect to H-scores for stathmin, pS6, pMTOR and pAKT are illustrated in Figure 2. Analysis of the H-scores in association with disease stabilization and AFP drop are detailed in Table 5. Only pMTOR was found to be associated with disease stabilization, 7 of the 10 patients (70%) who had high H-scores (> 20/300) achieved disease stabilization, in contrast to 7 out of 24 (29%) who had low H-scores (p = 0.028). The odds ratio (OR) for disease stabilization for high vs. low pMTOR H-scores is 5.667 (95% C.I. 1.129-28.454, p = 0.035).

**Table 4:** **Virological status, H-scores for stathmin, pS6, pMTOR and pAKT and clinical outcome in terms of having achieved disease stabilization of individual patient’s tumour**

Patient no.	HBV/HCV/ Non-B non-C	Stathmin	pS6	pMTOR	pAkt	Disease stabilization
PW004	HBV	High	Low	Low	High	No
PW005	HBV	High	High	Low	High	No
PW014	HBV	Low	High	Low	Low	No
PW018	HBV	High	Low	Low	Low	No
PW019	HBV	Low	High	Low	Low	No
PW020	Non-B, Non C	Low	High	Low	High	No
PW023	HCV	High	Low	High	Low	No
PW024	HBV	High	High	Low	High	No
PW025	HBV	High	Low	Low	High	No
PW028	HBV	High	Low	Low	High	No
PW029	Non-B, Non C	Low	Low	Low	Low	No
PW030	HBV	High	Low	Low	Low	No
PW031	HBV	High	Low	Low	Low	No
PW032	HBV	High	High	Low	Low	No
PW034	HBV	High	High	Low	High	No
PW035	Non-B, Non C	Low	Low	High	Low	No
PW036	HBV	Low	High	High	Low	No
PW039	HBV	High	Low	Low	Low	No
PW040	Non-B, Non C	High	High	Low	Low	No
PW042	HBV	High	Low	Low	Low	No
PW015	HBV	High	High	High	Low	Yes
PW016	HBV	High	High	Low	Low	Yes
PW017	Non-B, Non C	Low	Low	Low	Low	Yes
PW021	HBV	Low	Low	High	Low	Yes
PW022	Non-B, Non C	Low	Low	High	Low	Yes
PW026	HBV	Low	Low	Low	Low	Yes
PW027	HBV	High	High	Low	High	Yes
PW033	HBV	High	Low	High	High	Yes
PW037	HBV	Low	High	High	Low	Yes
PW038	HBV	High	High	Low	Low	Yes
PW043	HBV	High	High	High	High	Yes
PW044	HBV	Low	Low	Low	Low	Yes
PW045	HBV	Low	High	High	High	Yes
PW046	HBV	High	High	Low	Low	Yes

HBV- hepatitis B virus, HCV- hepatitis C virus, Non-B non-C- negative for hepatitis B or C.

Disease stabilization rate = (CR + PR + SD) >12 weeks.

**Table 5:** **Exploratory analysis on H-scores for stathmin, pS6, pMTOR and pAKT**

**Stathmin**			
H-scores Range: 0-300/300; Optimal Cut-off*: 15/300			
H-scores: High vs. Low	High H-scores (>15/300)	Low H-scores (≤15/300)	
Disease stabilization rate**	7/21 (33%)	7/13 (46%)	p = 0.238
OR for disease stabilization**	0.429 (95% CI 0.104-1.770)		p = 0.242
AFP response***	2/6 (33%)	6/16 (38%)	p = 0.376
**pS6**			
H-scores Range: 0-300/300; Optimal Cut-off*:120/300			
H-scores: High vs. Low	High H-scores (>120/300)	Low H-scores (≤120/300)	
Disease stabilization rate**	8/17 (47%)	6/17 (35%)	p = 0.489
OR for disease stabilization**	1.630 (95% CI 0.411-6.459)		p = 0.487
AFP response***	4/11 (36%)	4/11 (36%)	p = 0.341
**pMTOR**			
H-scores Range: 0-180/300; Optimal Cut-off*: 20/300			
H-scores: High vs. Low	High H-scores (>20/300)	Low H-scores (≤20/300)	
Disease stabilization rate**	7/10 (70%)	7/24 (29%)	p = 0.028
OR for disease stabilization**	5.667 (95% CI 1.129-28.454)		p = 0.035
AFP response	4/16 (25%)	4/6 (67%)	p = 0.085
**pAKT**			
H-scores Range: 0-240/300; Optimal Cut-off*: 5/300			
H-scores: High vs. Low	High H-scores (>5/300)	Low H-scores (≤5/300)	
Disease stabilization rate**	4/11 (36%)	10/23 (43%)	p = 0.693
OR for disease stabilization**	0.743 (95% CI 0.169-3.262)		p = 0.694
AFP response***	7/16 (44%)	1/6 (17%)	p = 0.215

*H-scores Optimal Cut-off based on ROC.

**Disease stabilization rate (CR + PR + SD) ≥12 weeks, number of patients available for analysis = 34; disease stabilization in association with H-scores were compared using Fisher’s exact and proportional hazard model.

***AFP response, number of patients available for analysis = 22; AFP drop in association with H-scores were compared using Fisher’s exact.

Of the 36 patients, 22 were eligible for AFP response; there were 8 AFP responders and 14 non-responders. Correlation study of AFP response with H-scores for stathmin, pS6, pMTOR and pAKT showed no association. Of interest, AFP response for high vs. low pMTOR scores occurred in 67% and 20% respectively (p = 0.085).

## Discussion

The present study confirmed the MTD for temsirolimus in patients with chronic liver disease and advanced HCC to be 25 mg weekly, which is the approved dose for metastatic renal cell carcinoma [9,10]. Common adverse reactions of temsirolimus noted in this study were consistent with the reported toxicity profile of this agent, which included skin and mucosal toxicities, constitutional symptoms (fatigue, anorexia, insomnia), myelosuppression, metabolic disturbances (disturbances in glucose and lipids controls) and the uncommon but well-known occurrence of interstitial pneumonitis.

In an unselected population of advanced HCC patients, the current study reveals that the use of temsirolimus yielded a 3-month PFS of 0.47, which is lower than the pre-specified limit considered to be efficacious. The present finding is in line with that of the EVOLVE study, in which everolimus has failed to achieve the primary endpoint in improving OS in an unselected HCC patient population who had progressed on sorafenib [19]. The discouraging result sheds light to the potential importance of suitable patient selection.

There has been limited ability to identify biomarkers for appropriate utilization of mTORIs. In the phase I study of everolimus, 11 HCC patients had pre-treatment tumour tissues available for assessment, one patient achieved PR and the tumour showed moderate to high levels of p-AKT, p-MTOR and pS6 [17]. The key effector in the PI3K/Akt/mTOR pathway is mTOR, which has a critical role in regulating cell proliferation, survival and angiogenesis [27,28]. PIK3CA has also been suggested as a predictive marker for effective mTOR inhibition in breast cancer [29,30], unfortunately, a recent report on endometrial cancer did not support this [31]. Further, the reported rate of mutations in the PIK3CA gene has been inconsistent in HCC varying from 0-35% [32,33]. Activated PI3K propels two downstream effectors: mTOR complex 2 (mTORC2) and Akt. Akt activates mTORC1 which in turn activates downstream effector, the serine/threonine kinase, S6K1. S6K1 participates in numerous cellular processes central to promoting cell proliferation, cell growth and cell cycle progression [34,35]. Phosphorylated mTOR and p-S6K is elevated in approximately 40% of HCC [6,27,36]. It has been observed that loss of PTEN, the negative regulator of PI3K, results in robust activation of this pathway [37,38], and stathmin, encoded by the signature gene STMN1, has been suggested to be a more accurate immunohistochemical marker of the PTEN signature [39]. These data have prompted us to explore the possibility of stathmin, pAKT, pMTOR and pS6 as potential biomarkers for response.

The present exploratory analyses show pMTOR to be the only marker associated with disease stabilization effect of temsirolimus. Although some studies suggested that pMTOR overexpression may have prognostic impact independent of temsirolimus, studies in different tumour types have reported conflicting results [40-42]. Specifically, a study in HCC patients undergoing orthotopic liver transplantation reported mTOR pathway to be active in 40% of the patients, but none of the biomarkers [PTEN, p-AKT, p-mTOR, p-p70S6K and p-4EBP-1] were associated with survival [43]. In this current study, assessment of pMTOR in relation to presence of vascular invasion and tumour grading was attempted; unfortunately, 22 of the 34 tumour analyzed were biopsy samples which limits detail pathological assessment.

On the other hand, the effect of rapalogs on Akt may vary with drug dose, with lower doses increasing Akt activation while higher doses diminishing Akt activity [44,45]. In addition, the effect on Akt also varies with cell type [46]. Thus, determining the clinical effects of different dosages of mTORIs could be an important tactic to overcoming such limitation.

Further, combining mTORIs with other systemic agents could improve clinical efficacy. The combination of everolimus and sorafenib has been reported to synergistically inhibit proliferation and tumor growth in HCC cell lines and xenografts [14]. A phase I study of this combination in advanced HCC patients yielded an encouraging 8% PR and 60% SD [47]. In addition, studies have shown that the activation of Akt markedly increases the resistance against microtubule-directed cytotoxic agents while mTORIs could inhibit this resistance [48,49].

## Conclusions

In summary, this study demonstrates that temsirolimus enables disease stabilization with tolerable toxicity profile among HCC patients. Although the efficacy data has not reached the pre-specified PFS endpoint, patients with tumours having a high pMTOR score were more likely to achieve disease stabilization. In this respect, a recent study among bladder cancer patients have reported that everolimus was more effective in patients with a somatic mutation in the TSC1 complex [50]. Therefore, the role pMTOR and TSC1 mutation as potential biomarkers for efficacy of mTOR inhibition should further be explored to enable better selection of appropriate patient population. However, further improvement in clinical efficacy for HCC will likely require combining mTORIs with other novel compounds.

## Abbreviations

HCC: Hepatocellular carcinoma
DLTs: Dose limiting toxicities
MTD: Maximum tolerated dose
CR: Complete response
PR: Partial response
SD: Static disease
PFS: Progression free survival
OS: Overall survival
mTORI: mTOR inhibitor
pMTOR: Phosphorylated mTOR
pS6K: Phosphorylated serine/threonine kinase
ROC: Receiver operating characteristic
AFP: Alfa-fetoprotein
